# Trait Anxiety Is Associated with Negative Interpretations When Resolving Valence Ambiguity of Surprised Faces

**DOI:** 10.3389/fpsyg.2016.01164

**Published:** 2016-08-03

**Authors:** Gewnhi Park, Michael W. Vasey, Grace Kim, Dixie D. Hu, Julian F. Thayer

**Affiliations:** ^1^Department of Psychology, Azusa Pacific University, AzusaCA, USA; ^2^The Ohio State University, ColumbusOH, USA; ^3^Rosemead School of Psychology, La MiradaCA, USA

**Keywords:** anxiety, negativity bias, surprised faces, spatial frequencies, interpretation bias

## Abstract

The current research examines whether trait anxiety is associated with negative interpretation bias when resolving valence ambiguity of surprised faces. To further isolate the neuro-cognitive mechanism, we presented angry, happy, and surprised faces at broad spatial frequency (BSF), high spatial frequency (HSF), and low spatial frequency (LSF) and asked participants to determine the valence of each face. High trait anxiety was associated with more negative interpretations of BSF (i.e., intact) surprised faces. However, the modulation of trait anxiety on the negative interpretation of surprised faces disappeared at HSF and LSF. The current study provides evidence that trait anxiety modulates negative interpretations of BSF surprised faces. However, the negative interpretation of LSF surprised faces appears to be a robust default response that occurs regardless of individual differences in trait anxiety.

## Introduction

It has been well documented that people with anxiety tend to interpret ambiguous stimuli negatively—termed *a negative interpretation bias* (Holmes et al., 2009). A negative interpretation bias has been consistently observed in anxious individuals in response to ambiguous words (Richards and French, 1992), sentences (Eysenck et al., 1991; MacLeod and Cohen, 1993), and scenarios (Hirsch and Mathews, 1997). For example, when an ambiguous sentence (e.g., “*The doctor examined little Emma’s growth*”) was presented, anxious individuals were more likely to choose a negative interpretation (e.g., “*The doctor looked at little Emma’s cancer*”) than a neutral interpretation (e.g., “*The doctor measured little Emma’s growth*”; Eysenck et al., 1991). As such, the anxiety-related negative interpretation bias has been investigated using words, sentences, and paragraphs, but relatively few studies have been conducted using facial expressions (Hallion and Ruscio, 2011). In the current research, we examined whether trait anxiety is associated with negative interpretation biases of surprised faces. Furthermore, we examined whether the anxiety-related interpretation bias depends on low level visual processing using different spatial frequency ranges.

### Cognitive Models of Anxiety-Related Interpretation Bias

The negative interpretation bias has been observed in both clinically anxious and high trait anxious individuals (Bishop, 2009). Furthermore, it has been suggested that the negative interpretation bias may play an important role in the etiology of a wide range of anxiety disorders (MacLeod et al., 2004, for a review; Wilson et al., 2006). Cognitive models of anxiety highlight competitive parallel processing and learning principles as possible mechanisms underpinning interpretation bias. For example, Mathews and Mackintosh (1998) suggest that there is a competition between negative and neutral representations activated by threat evaluation and top-down mechanisms, respectively (Bishop, 2009). However, high anxiety reinforces threat evaluation mechanisms to strengthen negative representations of ambiguous stimuli, which results in more negative interpretations. As a result, people with high-trait anxiety tend to make more negative interpretations, leading to frequent experiences of more intense anxious states (Wilson et al., 2006).

Human facial expressions provide important social and biological information and the ability to accurately interpret facial expressions may plays a critical role in navigating the social world and guiding their behavior (Ekman and Friesen, 1971; Adolphs, 2001; Marsh et al., 2005; Susskind et al., 2008; Park et al., 2012b). Particularly, people with high anxiety were hyper-sensitive to social cues, conveying threat (e.g., fearful or angry faces; Labuschagne et al., 2010). However, there are relatively few studies that have examined the anxiety-related negativity interpretation bias using facial stimuli. In one much-cited study, Richards et al. (2002) created morphed facial images by blending the two prototype emotional expressions (happiness, surprise, fear, sadness, disgust, and anger) in various proportions (e.g., 90% fear: 10% sadness, 70% fear: 30% sadness, 30% fear: 70% sadness, 10% fear: 90% sadness; Richards et al., 2002). High- and low-trait socially anxious people were presented with these morphed facial images and instructed to categorize each face (Richards et al., 2002). A morphed facial expression was presented on the screen until a verbal response was made. The result showed that high-trait socially anxious people classified face stimuli as being fearful more frequently than did low-trait socially anxious people. They were also slower in making responses for faces containing a proportion of happy expressions (Richards et al., 2002). However, they used the emotional identification task in which participants were instructed to classify emotional facial expressions. Thus, it is not clear whether people with anxiety exhibit deficiency in identifying specific emotional expressions or an interpretation bias in identifying expressions negatively or positively (Hallion and Ruscio, 2011). Also, they used artificial facial stimuli, instead of pictures of real faces which are ecologically more valid. In the current study, we took a more direct approach and asked participants to identify whether facial expressions had either a positive or negative valence. Particularly, we were interested in whether trait anxiety would be associated with negative and positive interpretation bias in response to surprised facial expressions.

### Emotional Ambiguity of Surprised Faces at Different Spatial Frequencies

Surprised facial expressions are unique. Some people interpret surprised facial expressions positively while others do so negatively (Neta and Whalen, 2010). In one neuroimaging study, people who made more negative interpretations of surprised faces showed increased activity in the amygdala (Kim et al., 2003, 2004). In contrast, people who made more positive interpretations of surprised faces showed reduced activity in the amygdala as well as increased activity in the ventromedial prefrontal cortex (vmPFC), which has been typically linked with regulatory function (Kim et al., 2003, 2004). By default, people initially make a negative interpretation of surprised faces—termed the *initial negativity hypothesis*; making a positive interpretation requires the exertion of regulation function to override the initial default response (Neta and Whalen, 2010).

A recent study revealed that negative interpretations of surprised facial expressions were even more pronounced when faces were presented at low spatial frequency (LSF; Neta and Whalen, 2010). Broad spatial frequencies (BSF) can be filtered to contain either HSF or LSF (e.g., Park et al., 2012b,c). LSF information is primarily conveyed via the magnocellular pathway involved in the rapid processing of depth, emotion, and low contrast black-and-white information (Livingstone and Hubel, 1988; Merigan and Maunsell, 1993; Vuilleumier et al., 2003; Nieuwenhuis et al., 2008; Park et al., 2012a,b,c). In particular, emotionally negative information (e.g., fearful faces) presented at low spatial frequencies are suggested to tap into the phylogenetically older *retinotectal* pathway which quickly conveys information from the retina through the superior colliculus and pulvinar nucleus of the thalamus to the amygdala (Livingstone and Hubel, 1988; Merigan and Maunsell, 1993; Vuilleumier et al., 2003; Nieuwenhuis et al., 2008; Park et al., 2012a,b,c). Thus, blurred and coarse LSF fearful faces elicited greater amygdala activity compared to LSF neutral faces (Vuilleumier et al., 2003).

High spatial frequency (HSF) information is primarily conveyed via the *parvocellular* pathway, which is associated with the processing of color and contrast information (Merigan and Maunsell, 1993; Vuilleumier et al., 2003; Park et al., 2012a,b,c). The parvocellular pathway has thin nerve fibers and transfers information rather slowly, but with high resolution (Merigan and Maunsell, 1993; Park et al., 2012a,b,c). Neuroimaging studies reported that fine and detailed HSF fearful faces elicited greater activation in ventral visual cortical areas, including the bilateral fusiform and the inferior temporal-occipital cortex (Vuilleumier et al., 2003; Winston et al., 2003; Park et al., 2012a,b,c). A recent study reported that people made more negative interpretations when surprised faces were presented at LSF than when presented at HSF (Neta and Whalen, 2010). In this study, we examined whether the anxiety-related negative interpretation bias would be observed in response to surprised faces.

However, it should be noted that whether high or LSF information is selectively processed via the parvocellular or magnocellular pathways, respectively, is still debatable. Skottun and Skoyles (2008), for example, mentioned that it was difficult to differentiate the two processing pathways via spatial frequency manipulations, if stimuli with high contrast are used. Furthermore, Pessoa and Adolphs (2010) pointed out that high as well as LSF filtered stimuli could thus be processed via the usual retino-cortical pathway. The amygdala could then be triggered based on cortical processing, not solely based on subcortical processing. In this vein, De Cesarei and Codispoti (2013) reviewed the studies and found that there were rather mixed results about the role that spatial frequency information played in emotional processing. Also, there were mixed reports on whether negative emotions at LSF elicited significantly greater activations in the amygdala (see also Morawetz et al., 2011). Despite that selective visual pathways associated with LSF and HSF information are debatable, converging evidence has shown that LSF information has been found to be more relevant for the processing of emotional information (Holmes et al., 2005; Bar et al., 2006; Laeng et al., 2010; Bannerman et al., 2012).

### The Present Study

The goal of the study was to examine whether anxiety is associated with negativity interpretation bias of surprised facial expressions, which may depend on different types of spatial frequencies. According to the initial negativity hypothesis, people initially make a negative interpretation of surprised faces (Kim et al., 2003, 2004; Neta and Whalen, 2010). We hypothesize that trait anxiety may modulate the interpretation of BSF surprised faces, such that people with high-trait anxiety may make more negative interpretations of BSF surprised faces. Trait anxiety may or may not modulate visual discrimination of LSF surprised faces. Previous research has shown that surprised facial expressions are rated even more negatively when they are presented at LSF (Neta and Whalen, 2010). With the synergistic effect of high trait anxiety, people with this trait may make even more negative interpretations of LSF surprised faces. Indeed, previous research indicates that people with high anxiety utilize more LSF information and less HSF information during visual perception of faces (Langner et al., 2009). It is also possible that trait anxiety may not modulate strong negative values associated with LSF surprised faces, considering that negative messages conveyed in LSF surprised faces are so robust that people may make negative interpretations regardless of anxiety levels. We hypothesize that trait anxiety is not related to the interpretation bias of HSF surprised faces because previous research has shown that people with high anxiety rely less on HSF information during face perception (Langner et al., 2009). In fact, people in general rely less on HSF information when discriminating emotions (Mermillod et al., 2008). However, it should be noted that the utilization of HSF information in the negative emotion detection was more appreciated, when participants were instructed to study the diagnosticity of information (Smith and Schyns, 2009). However, the current research did not encourage intentional processing. Thus, we did not expect that trait anxiety would modulate the effect of HSF on negativity bias.

We hypothesize that there is no difference between people with high and low trait anxiety in the interpretation of angry and happy faces at difference spatial frequencies. Research that investigated an interpretation bias has typically utilized ambiguous stimuli (e.g., ambiguous words, sentences, and scenarios) that require a certain degree of interpretation of information. However, explicitly negative or positive emotional stimuli do not require much interpretation: They are clearly perceived as emotionally negative or positive. However, it should be noted that there is a plethora of literature showing that anxious individuals exhibit attentional biases toward threat-relevant stimuli (Mathews et al., 1997; Cisler and Koster, 2010). For example, when schematic faces with ‘angry,’ ‘neutral,’ and ‘happy’ facial expressions were used as cues in the modified emotion spatial cueing task, high anxious individuals were slower to disengage their attention away from angry faces (Fox et al., 2002). However, there is almost no evidence supporting the perceptual bias favoring negative information.

## Materials and Methods

### Participants

Fifty-six undergraduate students successfully completed the study for partial course credit. The behavioral data from two participants were lost due to a computer error. All participants had normal or corrected to normal vision (20/20 visual acuity). People with a history of vision disorders or dysfunctions or neurological or psychiatric disorders were excluded from this experiment. We excluded data from one participant who showed non-normative ratings (e.g., rating angry as positive and happy as negative on greater than 45% of trials), yielding 53 participants (35 females; ages 18–25 years, mean age = 19 years). Participants received a written informed consent form prior to participating in the study, and all the experiments were reviewed and approved by the Azusa Pacific University Institutional Review Board (IRB): Approval number: #23-16.

### Stimuli

**FIGURE 1:**
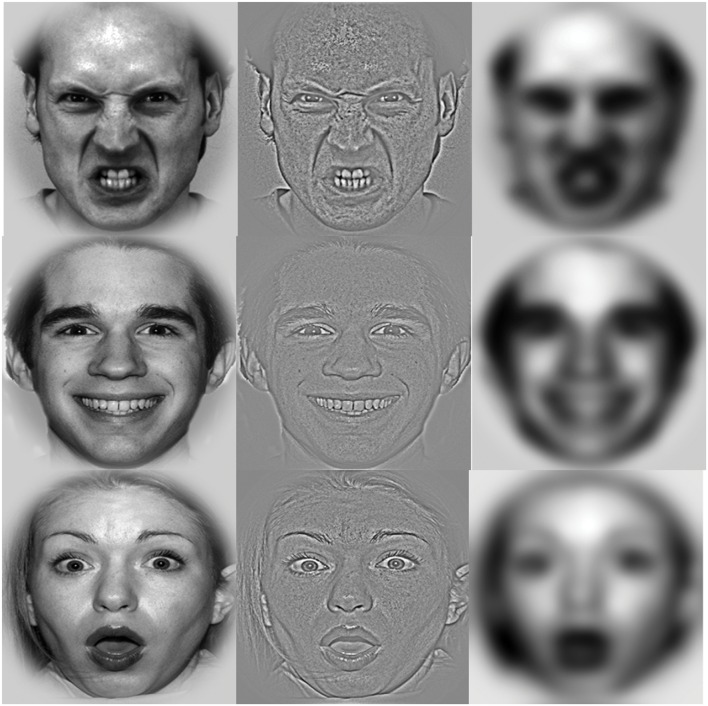
**Example stimuli.** Normal broad spatial frequency (BSF) angry, happy, and surprised faces (left column), high spatial frequency (HSF) faces (middle column), low spatial frequency (LSF) faces (right column).

We selected 66 faces (33 women and 33 men) from the NimStim set (Tottenham et al., 2009), the Pictures of Facial Affect (Ekman and Friesen, 1976), and Karolinska Directed Emotional Faces (KDEF) database (Lundqvist et al., 1998). Of the 66 images, some posed all three expressions (surprised, happy, or neutral), while others posed only one or two of the expressions. All faces were converted to gray-scale. Contrast and brightness were adjusted to maintain constancy across different face sets. As seen in **Figure 1**, each face was enclosed in a circular frame using Adobe PhotoShop CS3 software (Adobe System, San Jose, CA, USA) to exclude non-facial features (e.g., hair). In order to produce the HSF and LSF stimuli, the unfiltered (i.e., BSF) pictures were filtered through a high-pass cut off of >24 cycles/image for the HSF stimuli and a low-pass cut off of <6 cycles/image for the LSF stimuli. We used 198 faces (66 angry, 66 happy, and 66 surprised; 22 BSF, 22 HSF, and 22 LSF with each expression) for experimental trials and 12 faces for practice trials. Average gray-scale values for the BSF, HSF, and LSF stimuli were 135.74, 135.32, and 135.29, respectively, and for the angry, happy, and surprised face categories the average gray-scale values were 135.43, 135.20, and 135.63, respectively, on a 256 gray-level scale. These average gray-scale values did not significantly differ across spatial frequencies, *F*(2,130) = 0.99, *p* = 0.32, $\eta_{p}^{2}$ = 0.02, or emotional expression, *F*(2,130) = 0.45, *p* = 0.63, $\eta_{p}^{2}$ = 0.01. Each stimulus measured 6° horizontally and 6° vertically against a black background at a viewing distance of 65 cm and was displayed on a 17 inch LCD flat-panel monitor with a resolution of 1024 × 768 pixels.

### Procedure

All participants performed the valence task individually in a dimly lit room. The same identity was not presented as both BSF and filtered images because previous research indicated that being exposed to BSF images would influence the ratings of the filtered images (Vuilleumier et al., 2003; Neta and Whalen, 2010). Participants were told that they would be presented with a series of pictures of unfamiliar faces, and their task would be to identify the valence of each face by pressing the “1” key for positive and the “2” key for negative on a number pad with their dominant hand. Participants were presented with 12 practice trials, followed by 198 experimental trials in three blocks that consisted of 66 trials each. After each block, participants were allowed a short break. The order in which each bock was presented was counterbalanced across each participant. Each trial began with a fixation point for 500 ms, followed by the display with an image for 200 ms at the center of the screen on a black background. The interstimulus interval varied from 1800 to 5800 ms (*M* = 3800). Participants were instructed to respond as quickly and accurately as possible. Participants received a “No response” feedback when they failed to respond within 3000 ms. After the task, participants completed the Spielberger State-Trait Anxiety inventory (Spielberger et al., 1983).

## Results

To provide more direct information on valence, we obtained mean valence scores for each participant by subtracting the mean of negative responses from the mean of positive responses. We first sought to replicate the previous findings (Neta and Whalen, 2010). We conducted a 3 (Spatial Frequency: BSF, HSF, and LSF) × 3 (Emotional Expressions: angry, happy, and surprised) repeated measures ANOVA on the mean valence score (**Table 1**). As expected, a significant interaction between spatial frequency and emotional expressions on the mean valence score, *F*(4,208) = 34.79, *p* < 0.001, $\eta_{p}^{2}$ = 0.40 was found. More importantly, consistent with the previous study (Neta and Whalen, 2010), paired *t*-tests (2-tailed) showed that LSF surprised faces (*M* = -0.79, *SD* = 0.25) were rated more negatively compared to HSF surprised faces (*M* = -0.63, *SD* = 0.34), *t*(52) = -3.67, *p* < 0.01, *d* = 0.54, and BSF surprised faces (*M* = -0.52, *SD* = 0.33), *t*(52) = -6.08, *p* < 0.001, *d* = 0.96. Also, there was no difference between LSF angry and LSF surprised faces (*p* > 0.42).^1^ Furthermore, replicating the previous findings (Neta and Whalen, 2010), we found that BSF angry expressions were rated as more negative than LSF angry expression, *t*(52) = -3.50, *p* < 0.001, *d* = 0.68. HSF angry expression were rated as more negative than LSF angry expression, *t*(52) = -5.39, *p* < 0.001, *d* = 1.0. In addition, both BSF and HSF happy expressions were rated as more positive than LSF happy expressions, (*p*s < 0.001). The main effect of emotion was significant, *F*(2,104) = 1602.36, *p* < 0.001, $\eta_{p}^{2}$ = 0.97. *Post hoc* comparisons using the Tukey HSD test indicated that angry expressions (*M* = -0.89, *SD* = -0.08) were rated more negatively compared to happy (*M* = 0.89, *SD* = 0.12) and surprised expressions (*M* = -0.65, *SD* = -0.25) and that surprised expression were rated more negatively compared to happy expressions. Also, the main effect of spatial frequency was significant, *F*(2,104) = 15.01, *p* < 0.001, $\eta_{p}^{2}$ = 0.22. *Post hoc* comparisons using the Tukey HSD test indicated that LSF (*M* = -0.29, *SD* = -2.10) were rated more negatively compared to BSF (*M* = -0.21, *SD* = -1.50) and HSF (*M* = -0.17, *SD* = -1.20), but there was no difference between BSF and HSF.

**Table 1:** Mean valence scores as a function of spatial frequency and emotional expressions.

Conditions	Mean valence (*SD*)
BSF angry faces	-0.92 (0.15)
HSF angry faces	-0.93 (0.09)
LSF angry faces	-0.82 (0.11)
BSF happy faces	0.94 (0.09)
HSF happy faces	0.94 (0.09)
LSF happy faces	0.74 (0.27)
BSF surprised faces	-0.52 (0.33)
HSF surprised faces	-0.63 (0.34)
LSF surprised faces	-0.79 (0.25)


*Standard deviations in parenthesis.*

### Analysis of Mean Valence Score and Trait Anxiety

**FIGURE 2:**
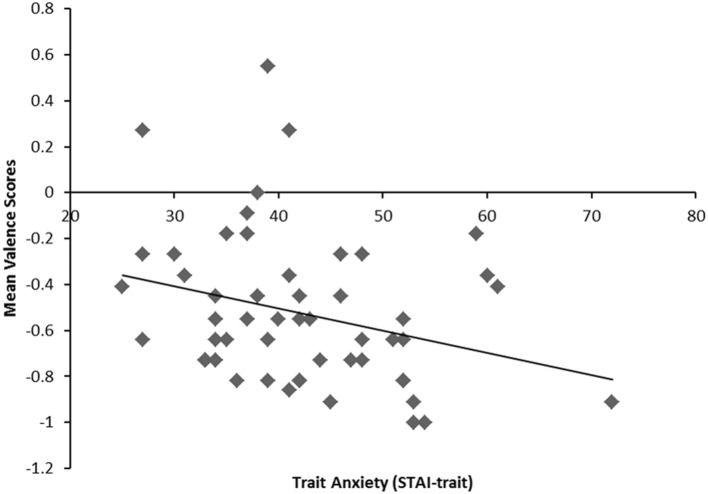
**A scatterplot indicating the negative correlation between trait anxiety (*x*-axis) as a continuous measure and mean valence scores to BSF surprised faces (*y*-axis).** *r* = 0.29, *p* = 0.04.

We predicted that people with high trait anxiety would interpret BSF surprised faces more negatively compared to people with low trait anxiety. However, it was unclear whether trait anxiety would modulate negativity bias in response to LSF surprised faces. We expected that trait anxiety would not modulate the interpretation bias of HSF surprised faces. To assess the effects of trait anxiety on negativity ratings, we conducted a 3 (Spatial Frequency: BSF, HSF, and LSF) × 3 (Emotional Expressions: angry, happy, and surprised) repeated measures analysis of covariance (ANCOVA) with z-standardized trait anxiety (STAI-trait) and state anxiety (STAI-state) scores as covariates on mean valence scores. There were significant main effects of emotion, *F*(2,100) = 1625.20, *p* < 0.001, $\eta_{p}^{2}$ = 0.97 and spatial frequency, *F*(2,100) = 16.57, *p* < 0.001, $\eta_{p}^{2}$ = 0.25. These main effects were qualified by the significant two-way interaction between spatial frequency and emotion, *F*(4,200) = 36.90, *p* < 0.001, $\eta_{p}^{2}$ = 0.43, which was also qualified by three-way interaction between spatial frequency, emotion, and trait anxiety, *F*(4,200) = 3.77, *p* < 0.01, $\eta_{p}^{2}$ = 0.07.^2^ To decompose the interaction, we examined the main effects and interaction between spatial frequency and trait anxiety for angry, happy, and surprised facial expressions, separately. With surprised facial expressions, there was a significant interaction between trait anxiety and spatial frequency, *F*(2,100) = 7.88, *p* < 0.01, $\eta_{p}^{2}$ = 0.14. As predicted, trait anxiety was negatively correlated with the mean valence scores of BSF surprised faces, *r* = -0.29, *p* < 0.04 (2-tailed; see **Figure 2**). However, there was no significant relationship between trait anxiety and BSF angry or happy faces, (*p*s > 0.32). Therefore, consistent with our predictions, participants with high anxiety gave significantly more negative ratings for BSF surprised faces.

However, at HSF and LSF, there was no interaction between trait anxiety and emotional expressions (*p*s > 0.18). Thus, as predicted, trait anxiety was not related to the ratings of HSF surprised faces. We did not offer a specific hypothesis for LSF surprised faces. The results revealed that trait anxiety did not modulate the ratings for LSF surprised faces.

## Discussion

The current study investigated whether trait anxiety is associated with negative interpretations when resolving the valence ambiguity of surprised faces. Replicating the previous finding (Neta and Whalen, 2010), LSF surprised faces were rated more negatively compared to HSF surprised faces and BSF surprised faces. As predicted, trait anxiety was negatively associated with mean valence scores of BSF surprised faces, but not with HSF surprised faces. We did not offer a specific hypothesis for LSF surprised faces. The results evinced that trait anxiety did not modulate the rating of LSF surprised faces.

Previous research has shown that when ambiguous words (Richards and French, 1992), sentences (Eysenck et al., 1991; MacLeod and Cohen, 1993), and scenarios (Hirsch and Mathews, 1997) are presented, people with high anxiety tend to interpret them negatively (Holmes et al., 2009). Our results provide additional evidence that people with high-trait anxiety are more likely to negatively interpret surprised faces. As such, it appears that individual differences in trait anxiety play an important role in determining the valence ambiguity of BSF surprised faces.

The results of the current study provide a converging line of evidence for the initial negativity hypothesis (Neta and Whalen, 2010). According to the initial negativity hypothesis (Neta and Whalen, 2010), a default interpretation of surprised faces is negative, and LSF information facilitates a negative interpretation. In fact, several researchers have argued that LSF information in emotional stimuli is processed rapidly, but coarsely, by the phylogenetically old *retinotectal* pathway which conveys information through the superior colliculus and pulvinar nucleus of the thalamus to the amygdala (Livingstone and Hubel, 1988; Merigan and Maunsell, 1993; Vuilleumier et al., 2003; Palermo and Rhodes, 2007; Nieuwenhuis et al., 2008; Park et al., 2013). Indeed, LSF information in surprised faces may facilitate the default threat response by directly tapping into the amygdala and subcortical mechanisms of face perception. Our results extend the previous finding and suggest that the negative interpretation of LSF surprised faces is a robust phenomenon that cannot be modulated by trait anxiety.

It should be noted that perceptual processing of different spatial frequency information may have in part contributed to the negative interpretation of LSF surprised faces. We found that LSF angry expressions were rated as less negative and LSF happy expressions as less positive scores compared to BSF information. The results appear to suggest that it was more difficult to perceive LSF information in general. However, perceptual difficulty does not fully account for the results. If the results are mainly due to perceptual difficulty associated with LSF information, we would expect that there is relatively little perceptual difference across different emotions at LSF information. The results showed that there were significant differences between LSF angry and LSF happy faces and between LSF surprised and LSF happy faces; however, there was no difference between LSF angry and LSF surprised faces. Thus, it may be reasonable to argue that perceptual difficulty associated with LSF information may have contributed to negative bias associated with LSF surprised faces, but does not fully account for our results.

It is possible that high-trait anxiety individuals with strong attentional control may override the initial tendency to make negative interpretations and make positive interpretations. A number of studies showed that attentional control plays an important role in anxiety-related attentional bias favoring negative stimuli. Derryberry and Reed (2002, p. 226) defined effortful control (EC) as “a self-regulatory dimension in relation to more reactive dimensions of positive emotionality and negative emotionality.” EC constrains overly reactive emotions and plays a significant role in disengaging from threatening cues and engaging in safety cues (Park, 2009). According to Derryberry and Reed (2002), anxious individuals with poor EC exhibited attentional biases favoring threatening stimuli, whereas anxious individuals with good EC were capable of shifting their attention away from threatening stimuli and engaging in safety stimuli. Lonigan and Vasey (2009) also demonstrated the interaction between negative affectivity (NA) and EC on attentional biases favoring negative information in children. Therefore, it is possible that high-trait anxiety individuals with good EC may override the default negativity bias and make positive interpretations of surprised faces. Unfortunately, we did not measure effort control of participants, but this may be a fruitful avenue for future research.

The limitation of the study is that button press has not been counterbalanced (i.e., participants always responded “1” for positive and “2” for negative); thus we cannot completely rule out the possibility that bias in motor control may have contributed to the result. Secondly, although we used the stimuli that are already validated, we did not measure the emotion recognition rate of each stimulus.

## Conclusion

We examined whether trait anxiety modulated negative interpretation biases of surprised faces. As expected, high trait anxiety was associated with more negative interpretations of BSF surprised faces. However, anxiety modulation disappeared at LSF surprised faces. The current study provides strong support for the initial negativity hypothesis, which is more prominent in people with high trait anxiety. Furthermore, the results of this study provide evidence that negative interpretations of LSF surprised faces may be common default interpretations that occur regardless of individual differences in trait anxiety.

## Author Contributions

GP designed the experiments and collected data with GK. GP analyzed the data and wrote the manuscript with critical edits from MV, DH, and JT.

## Conflict of Interest Statement

The authors declare that the research was conducted in the absence of any commercial or financial relationships that could be construed as a potential conflict of interest.

## Footnotes

1 However, there were significant differences between LSF angry and LSF happy faces, *t*(52) = -41.55, *p* < 0.001, and between LSF surprised and LSF happy faces, *t*(52) = 33.68, *p* < 0.001.

2 We also computed the percentage of negative ratings, identical to Neta and Whalen (2010), and conducted a 3 (Spatial Frequency: BSF, HSF, and LSF) × 3 (Emotional Expressions: angry, happy, and surprised) × 2 (Trait Anxiety Level: high, low) mixed factorial analysis of covariance (ANCOVA) with z-standardized trait anxiety (STAI-trait) and state anxiety (STAI-state) scores as covariates. Consistently, the three-way interaction between spatial frequency, emotion, and anxiety was significant, *F*(4,200) = 3.14, *p* < 0.01, $\eta_{p}^{2}$ = 0.06.
